# Quantification Accuracy of Liver Fibrosis by *In Vivo* Elastography and Digital Image Analysis of Liver Biopsy Histochemistry

**DOI:** 10.1155/2014/317635

**Published:** 2014-12-23

**Authors:** Justinas Besusparis, Skirmante Jokubauskiene, Benoit Plancoulaine, Paulette Herlin, Aida Laurinaviciene, Arida Buivydiene, Arvydas Laurinavicius

**Affiliations:** ^1^Department of Pathology, Forensic Medicine and Pharmacology, Faculty of Medicine, Vilnius University, 21 M. K. Ciurlionio Street, LT-03101 Vilnius, Lithuania; ^2^Université de Caen, Esplanade de la Paix, 14032 Caen, France; ^3^Centre of Hepatology, Gastroenterology and Dietetics, Vilnius University Hospital Santariskiu Clinics, 2 Santariskiu Street, LT-08661 Vilnius, Lithuania; ^4^Clinic of Gastroenterology, Nephrourology and Surgery, Medical Faculty, Vilnius University, 2 Santariskiu Street, LT-08661 Vilnius, Lithuania

## Background

Chronic hepatitis C is a rapidly spreading infection and continues to be leading cause of chronic liver disease. Liver fibrosis staging is essential in management on these patients. The accurate assessment of hepatic fibrosis plays an important role for determining treatment, screening strategies, and prognosis. The aim of this study was to evaluate accuracy of noninvasive transient elastography and three digital image analysis tools, for measuring the extent of fibrosis in human liver biopsies, based on the biopsy fibrosis reference data obtained by stereological point counting method.

## Methods

Liver biopsy cores from 68 patients diagnosed with viral hepatitis C were used in this study. Total liver fibrosis was evaluated by transient elastography, digital image analysis on Masson's trichrome (MAS), and Picro-Sirius Red (PCR) stained tissue specimens, using Leica/Aperio Colocalization, Genie image analysis software, and a home-made principal component analysis algorithm (PCA). Stereology grid count (20 out of 68 digital slides) and pathologist's visual score using METAVIR grading system were performed. Stereological estimation of the volume fraction of fibrosis was taken as a reference. All methods were compared, using correlation, linear regression analysis, and ANOVA test.

## Results

The volume fractions of fibrosis obtained by PCR Colocalization (8.91 ± 4.73) were closest to the reference value of PCR estimated by stereology (10.78 ± 7.46) while other methods indicated underestimation. The PCR stereology values correlated strongly with the values obtained using the Colocalization (*r* = 0.95, *P* < 0.001) and Genie (*r* = 0.98, *P* < 0.001) software. Single linear regression analysis demonstrated some advantage of the PCR Genie analysis over the PCR Colocalization, transient elastography, and PCA. In log-transformed measurements for *r*-square 0.96 the slope was 0.925 for PCR Genie versus *r*-square 0.91 with a slope of 0.986 for PCR Colocalization, *r*-square 0.76 with a slope of 0.42 for PCA, and *r*-square 0.35 with a slope of 0.917 for elastography. ANOVA revealed statistically significant differences of PCR Colocalization and PCR Genie results between METAVIR II, III, and IV groups, including pairwise comparisons, except for I versus II groups.

## Conclusion

Digital analysis methods applied to Picro-Sirius Red histochemical staining of biopsy material revealed almost perfect correlation with criterion standard obtained by stereology point counting and outperformed Masson's trichrome staining and transient elastography. PCR Genie algorithm could be the method of choice with a slight underestimation bias, which is considered acceptable for both clinical and research purposes.

